# Co‐existence of Distinct Supramolecular Assemblies in Solution and in the Solid State

**DOI:** 10.1002/chem.201604832

**Published:** 2016-12-22

**Authors:** G. N. Manjunatha Reddy, Aida Huqi, Dinu Iuga, Satoshi Sakurai, Andrew Marsh, Jeffery T. Davis, Stefano Masiero, Steven P. Brown

**Affiliations:** ^1^ Department of Physics and Department of ChemistryUniversity ofWarwick, CoventryCV4 7ALUK; ^2^ Dipartimento di Chimica “Giacomo Ciamician”, Alma Mater StudiorumUniversità di Bologna40126BolognaItaly; ^3^ JEOL (UK), Silver Court, WatchmeadWelwyn Garden CityAL7 1LTUK; ^4^ Department of Chemistry and BiochemistryUniversity of MarylandCollege ParkMD20742USA

**Keywords:** 2D double-quantum spectroscopy, fast magic-angle spinning, NMR spectroscopy, self-assembly, supramolecular chemistry

## Abstract

The formation of distinct supramolecular assemblies, including a metastable species, is revealed for a lipophilic guanosine (G) derivative in solution and in the solid state. Structurally different G‐quartet‐based assemblies are formed in chloroform depending on the nature of the cation, anion and the salt concentration, as characterized by circular dichroism and time course diffusion‐ordered NMR spectroscopy data. Intriguingly, even the presence of potassium ions that stabilize G‐quartets in chloroform was insufficient to exclusively retain such assemblies in the solid state, leading to the formation of mixed quartet and ribbon‐like assemblies as revealed by fast magic‐angle spinning (MAS) NMR spectroscopy. Distinct N−H⋅⋅⋅N and N−H⋅⋅⋅O intermolecular hydrogen bonding interactions drive quartet and ribbon‐like self‐assembly resulting in markedly different 2D ^1^H solid‐state NMR spectra, thus facilitating a direct identification of mixed assemblies. A dissolution NMR experiment confirmed that the quartet and ribbon interconversion is reversible–further demonstrating the changes that occur in the self‐assembly process of a lipophilic nucleoside upon a solid‐state to solution‐state transition and vice versa. A systematic study for complexation with different cations (K^+^, Sr^2+^) and anions (picrate, ethanoate and iodide) emphasizes that the existence of a stable solution or solid‐state structure may not reflect the stability of the same supramolecular entity in another phase.

## Introduction

**Figure 1:**
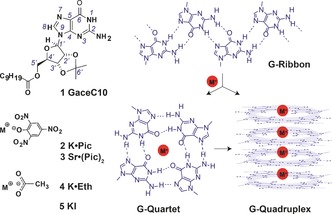
2′,3′‐*O*‐isopropylidene‐5′‐decanoylguanosine (GaceC10, **1**), potassium picrate (K⋅Pic, **2**), strontium picrate (Sr⋅(Pic)_2_, **3**), potassium ethanoate (K⋅Eth, **4**) and potassium iodide (KI, **5**), together with a schematic of G‐quadruplex and G‐ribbon assemblies.

The transition from solution‐ and gel‐phases to the solid state, or vice versa, is a key step in generating functional supramolecular assemblies,^1^ for example, by using relatively simple building blocks such as nucleobases.^2^ In this bottom‐up synthetic approach, characterization of assembly–disassembly pathways including the identification of intermediates and metastable species is a crucial aspect which allows a better formulation of structure‐function relationships for such materials with the aim of improving the design process.^3^ The existence of supramolecular structures made by nucleobase building blocks is neatly exemplified by X‐ray diffraction studies,^4^ electron microscopy and modelling approaches,^5^ as well as solution and solid‐state NMR spectroscopy techniques.^6^ However, the problem of understanding the hierarchy of self‐assembly becomes more challenging when different assemblies co‐exist either due to the nature of the system or the reaction conditions that lead to the formation of supramolecular aggregates. To this end, it remains to be established how molecular constituents self‐assemble into ordered supramolecular structures in solution, gel and in the solid state, such as those exhibited by guanosine (G)‐based systems (Figure 1, quartet and ribbon‐like structures), which have a wide variety of applications ranging from chemical biology^7^ to soft matter and organic electronics.^8^

Cation‐templated G‐quartets (G4) are central to many biomedical applications,^7^ as well as enabling supramolecular chemists to build functional architectures^8^ such as gelators,^9^ membrane films,^10^ nanowires,^11^ synthetic ionophores and ion channels,^12^ and to achieve separation for rare earth metals and radioactive isotopes.^13^ Interestingly, the formation and stability of G4 assemblies is notably different for various G‐derivatives,3d, 8, 9, 11, 12, 14 including a case for which the formation of a G‐quartet occurs without a templating cation.^15^ Moreover, for this class of lipophilic guanosine (G) derivatives,^8^ interconversions between different G‐assemblies can be controlled by cation or anion complexation.^14^

Our studies herein reveal that the formation of a lipophilic G‐quadruplex is remarkably sensitive to a solution‐ to solid‐state transition, or vice versa, even in the presence of excess K^+^ ions, which stabilize such assemblies in weakly polar organic solvents such as chloroform. A series of 2^′^,3^′^‐*O*‐isopropylidene‐5^′^‐decanoylguanosine (GaceC10, **1**) complexes were prepared using different proportions of potassium picrate (K⋅Pic, **2**), strontium picrate (Sr⋅Pic_2_, **3**), potassium ethanoate (K⋅Eth, **4**) and potassium iodide (KI, **5**) salts (Figure 1) with the aim of understanding: 1) processes leading to the formation of G4 assemblies in solution and in the solid state in the presence of at least 0.125 equivalents of potassium ions (i.e., a 8:1 ratio corresponding to one cation shared between two G‐quartets); 2) the role of cation and anion binding for stabilizing these G4 assemblies in solution and in the solid state; 3) the reversibility of solid–solution interconversions including the identification of metastable intermediates.

## Experimental Section

Reagents, solvents and NMR tubes (5 mm o.d., suitable for >100 MHz instruments) were purchased from Sigma–Aldrich, Gillingham, UK and used as received, except where otherwise stated. Potassium and strontium picrates^16^ and 2′‐3′‐isopropylideneguanosine^17^ were prepared according to previously described procedures, or purchased from Sigma–Aldrich. Precautions were taken during all the synthetic operations to prevent contact with potential sources of alkali metal ions: thus, chromatography on silica gel was avoided and Millipore water was used for washings. Melting points were recorded using a Stuart Scientific SMP10 apparatus. Elemental analysis measurements were made by Warwick Analytical Service Limited using a CE440 Analyzer. Infrared spectra were recorded using a Bruker Alpha spectrometer with a diamond attenuated total reflection device. For circular dichroism (CD) experiments, 0.65 mm solution GaceC10⋅M^+^A^−^ complexes were prepared in spectroscopic grade chloroform. All CD spectra were recorded using a Jasco J‐715 instrument and a 1 mm path length cuvette.

### Solution‐state NMR spectroscopy

For GaceC10 dissolved in [D_6_]DMSO, solution‐state NMR spectra were recorded with a 600 MHz Varian Inova instrument and referenced to residual solvent peaks. For GaceC10 dissolved in [D]chloroform (16 millimolar), spectra were recorded with either a Bruker Avance III HD 400 MHz or a Bruker Avance III 500 MHz spectrometer, with 32 transients co‐added. For GaceC10⋅K⋅Pic 8:2 and 8:4 complexes (32 mm in [D]chloroform), time course NMR data were acquired on a Jeol JNM‐ECZR 500 MHz spectrometer: NMR data acquisition was initiated immediately after the dissolution and monitored, overnight. 32 transients were co‐added. Temperature control was set to 298 K during the time course NMR data acquisition. In all cases, a recycle delay of 2 s was used.

### Powder X‐ray diffraction (PXRD)

For both GacC10 and a GaceC10⋅K⋅Pic 8:1 complex, PXRD data were collected at room temperature on a PANalytical X′Pert Pro MPD (Kα_1_ *λ*=1.5406 Å) equipped with monochromatic Cu Kα_1_ radiation and a PIXcel detector.

### Solid‐state NMR spectroscopy

Powdered solids were prepared by solvent evaporation (see section 1b of the Supporting Information for further details). For a GaceC10⋅K⋅Pic (8:1) complex, solid‐state to solution‐state transformation experiments were performed using Bruker 1.3 mm rotors wherein silicon spacers were used to ensure the mixed solid‐solution sample remained in the rotor during fast MAS experiments. Otherwise, 0.8 mg of the GaceC10 complex was packed into a JEOL 1.0 mm (outer diameter) rotor capable of achieving MAS frequencies up to 75 kHz. Solid‐state NMR experiments were performed at room temperature using a 20 T (^1^H Larmor frequency, 850 MHz) Bruker Avance III spectrometer equipped with either a JEOL 1 mm HX probe (tuned into double resonance mode) for 75 kHz MAS experiments or otherwise a Bruker 1.3 mm HXY probe (tuned into double resonance mode). ^1^H one‐pulse spectra were recorded by acquiring 128 co‐added transients using a recycle delay of 2 s. In all cases, the ^1^H shifts are calibrated with respect to neat TMS using adamantane as an external reference at 1.85 ppm.^18^

^1^H Double‐quantum (DQ) NMR spectra were recorded by using one rotor period of the BABA^19^ (Back to Back) recoupling sequence for the excitation and reconversion of DQ coherence. A nested 16‐step phase cycle was used in order to select Δ*p*=±2 on the DQ excitation pulses (4 steps) and Δ*p*=−1 (4 steps) on the *z*‐filter 90° pulse, whereby *p* is the coherence order. 512 *t*_1_ FIDs, each with 32 co‐added transients, were acquired using the States method to achieve sign discrimination in the *F*_1_ dimension with a rotor‐synchronized *t*_1_ increment of 13.3 μs, corresponding to an overall experimental time of 9.1 h using a 2 s recycle delay.

^1^H NOESY‐like spin diffusion (SD) MAS NMR spectra were recorded by using a rotor synchronized (in *t*_1_) three‐pulse sequence using the States method to achieve sign discrimination in the *F*_1_ dimension. A nested 16‐step phase cycle was used in order to select Δ*p*=±1 coherences on the excitation 90° pulse (2 steps) and the mixing 90° pulse (2 steps) and Δ*p*=−1 (4 steps) on the read‐out 90° pulse, in which *p* is the coherence order. For each mixing time, 512 *t*_1_ FIDs, each with 16 co‐added transients, were acquired using a rotor‐synchronized *t*_1_ increment of 13.3 μs, corresponding to an overall experimental time of 4.6 h using a 2 s recycle delay.

## Results and Discussion

### Combined solution‐ and solid‐state studies confirm the formation of distinct supramolecular assemblies

**Figure 2:**
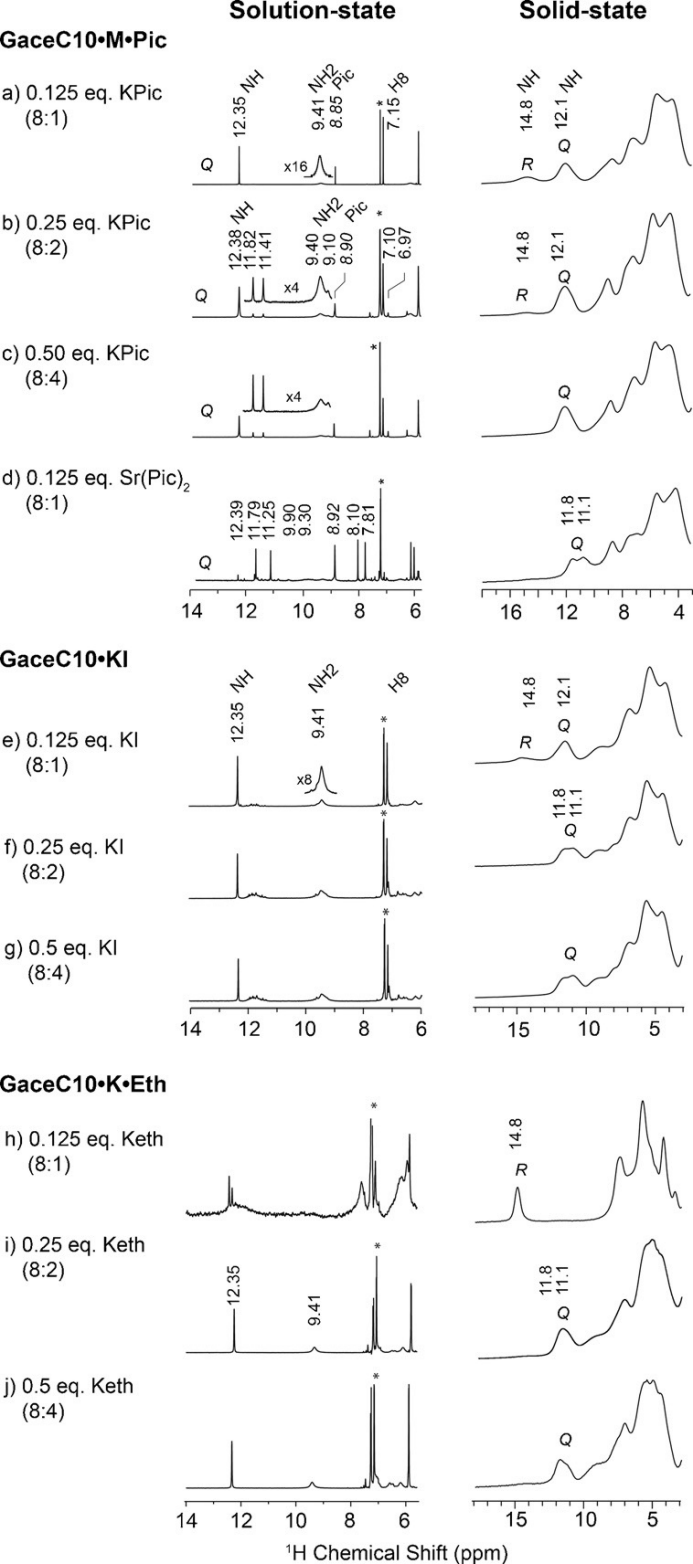
One‐pulse ^1^H solution‐state (left, 400 MHz, 8 mm in CDCl_3_) and solid‐state (right, 850 MHz, 75 kHz MAS) NMR spectra of GaceC10⋅K⋅Pic a) 8:1, b) 8:2 and c) 8:4 complexes, d) a GaceC10⋅Sr⋅(Pic)_2_ 8:1 complex; GaceC10⋅KI, e) 8:1, f) 8:2, g) 8:4 complexes; and GaceC10⋅K⋅Eth h) 8:1, i) 8:2, and j) 8:4 complexes. Chemical shifts corresponding to NH, NH2 and H8 (the aromatic CH) protons in GaceC10 and to picrate are noted, while the asterisk indicates the residual chloroform peak–note that the ^1^H solution‐state chemical shifts for c) GaceC10⋅K⋅Pic 8:4 are the same as for b) GaceC10⋅K⋅Pic 8:2. Q and R denote quartet and ribbon‐like assembly, respectively, with a Q/R ratio wt/wt (±5), 65:35 in (a), 84:16 in (b) and 64:36 in (e) as measured by a line shape fitting analysis of the NH peaks (Supporting Information, Figure S5).

**Figure 3:**
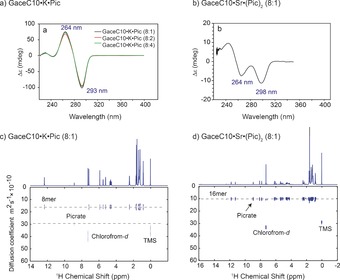
CD spectra (top) in chloroform, 0.65 mm, for: a) GaceC10⋅K⋅Pic and b) GaceC10⋅Sr⋅(Pic)_2_ complexes. ^1^H (500 MHz) DOSY NMR spectra (bottom) of c) GaceC10⋅K⋅Pic, and d) GaceC10⋅Sr⋅(Pic)_2_ 8:1 complexes, 16 mm in CDCl_3_.

The formation of hydrogen bonded assemblies in solution depends on equilibria involving timescales that vary between milliseconds and months.14a,14e For the GaceC10⋅K^+^ complexes dissolved in [D]chloroform, ^1^H NMR peaks in the vicinity of 12.35 and 9.41 ppm (e.g., Figure 2 a, e, f, g, i and j) are assigned to inter‐quartet Hoogsteen hydrogen bonded NH and NH_2_ protons, respectively.12b, 14e Multiple NH peaks are observed for K⋅Pic 8:2 and 8:4 complexes (Figure 2 b and c) immediately after the dissolution indicating the formation of distinct quartet‐based assemblies. However, the minor species (*δ*_NH_, 11.41 and 11.82 ppm, 1:1) are kinetically labile and slowly dissociated into the major (*δ*_NH_, 12.38 ppm) species within 8 h, as monitored by time‐course NMR spectroscopy (Supporting Information, Figure S2). For the other complexes, the ^1^H NMR spectra remained unchanged when monitoring over several days. Circular dichroism (CD) data show the formation of G‐quadruplexes^20^ with absorbance centred at 264 nm, although distinct CD spectral patterns are observed for K⋅Pic and Sr⋅(Pic)_2_ 8:1 complexes signifying the formation of different G4 assemblies (Figures 3 a, b and Supporting Information, Figure S3).

A pulsed field gradient (PFG) NMR diffusion‐ordered spectroscopy (DOSY) analysis of NH peaks showed measurably different diffusion constants for K⋅Pic and Sr⋅(Pic)_2_ complexes (see Table 1 and Figure 3 c and d).14e, 20a, 21 Changes in the measured diffusion coefficients (*D*) also reveal a variation in the binding affinity of picrate ions, that is, picrate binds weakly to GaceC10⋅K^+^ complexes and strongly to the GaceC10⋅Sr^2+^ complex. On the basis of the ^1^H NMR spectra shown in Figure 2 and the CD^20^ and PFG NMR14e, 20a data (see Figures 3, Table 1, and Supporting Information Figures S2 and S3), we propose the following: GaceC10⋅K⋅Pic and Sr⋅(Pic)_2_ 8:1 complexes form octamer (a single NH peak) and hexadecamer (two NH peaks in a 1:1 ratio, arising from outer and inner G‐quartets) species, respectively.12b, 14a,14e For the GaceC10⋅K⋅Pic 8:2 and 8:4 complexes, DOSY NMR analysis of the multiple NH peaks between 11 and 13 ppm (see Table 1 and Figure S2) showed nearly identical *D* values, suggesting the formation of *C*_4_ symmetric octamers as minor species (two NH peaks in a 1:1 ratio) which then slowly rearrange into *D*_4_ symmetric octamers (a single NH peak).

**Table 1:** Diffusion coefficients (*D*) of GaceC10 complexes measured by PFG NMR in [D]chloroform (see Figures 3 and S2).^[a]^

GaceC10⋅M⋅Pic complex	Component	*D*×10^−10^ m^2^ s^−1^ immediately after dissolution	*D*×10^−10^ m^2^ s^−1^ after 8 h
GaceC10⋅K⋅Pic (8:1)	8mer	16.9±1.1	–
	picrate	31.5±1.8	–
GaceC10⋅K⋅Pic (8:2)	8mer	17.0±1.2	18.3±1.5
		19.2±1.8	–
	picrate	47.9±2.5	41.6±0.2
GaceC10⋅K⋅Pic (8:4)	8mer	18.9±0.6	16.3±0.7
		20.7±1.5	–
	picrate	31.6±0.6	19.1±0.7
GaceC10⋅Sr⋅(Pic)_2_ (8:1)	16mer	9.9±0.03	–
	picrate	10.1±0.04	–

[a] We note that the results herein for GaceC10 complexes dissolved in [D]chloroform exhibit slightly higher diffusion coefficients (*D*, between 10 and 21×10^−10^ m^2^ s^−1^) as compared to the *D* values reported by Davis and co‐workers,20a and Meijer and co‐workers14e for differently substituted *t*Bu(Me)_2_Si‐G derivatives (*D*, between 2 and 4×10^−10^ m^2^ s^−1^) dissolved in highly polar solvents such as [D_3_]acetonitrile, [D_8_]THF and [D_6_]acetone.

### Fast MAS NMR reveals the presence of quartet and ribbon‐like assemblies in the solid state

Next to the corresponding ^1^H solution‐state NMR spectra, Figure 2 also presents ^1^H fast magic‐angle spinning (MAS, 75 kHz) solid‐state NMR spectra of dried powders prepared by evaporation from the corresponding solution (see Supporting Information, section 1 b, for further details). Although the resolution in the solid‐state NMR spectra is much poorer than in solution, distinct peaks can be resolved, such that clear differences between the solution‐ and solid‐state NMR high ppm chemical shifts can be identified. Moreover, the solid‐state ^1^H NMR spectra show evident changes that depend on the concentration and nature of the ionic species. Specifically, solid‐state ^1^H NMR spectra of GaceC10⋅K⋅Pic 8:1 and 8:2 and GaceC10⋅K⋅I 8:1 complexes (Figures 2 a, b and e) exhibit peaks at 12.1 and 14.8 ppm that are characteristic of both quartet and ribbon‐like assembles, respectively: this is investigated further by two‐dimensional solid‐state MAS NMR spectroscopy (vide infra). A line shape fitting analysis of one‐pulse NMR spectra quantified the Quartet:Ribbon (Q:R) ratios as: 65:35, 84:16 and 64:36 (error estimated as ±5 %) wt/wt for the GaceC10⋅K⋅Pic 8:1, 8:2 and KI 8:1 complexes, respectively (Supporting Information, Figure S5). Note that for the GaceC10⋅K⋅ Eth 8:1 complex (Figure 2 h), in which only ribbon‐like self‐assembly is observed in the solid state, there is a clear broadening at ≈12 ppm that we assign to ribbon‐like self‐assembly also in solution.

The ^1^H double‐quantum (DQ) single‐quantum (SQ) MAS NMR experiment is a powerful probe of proton‐proton proximities in the solid state.^22^ Specifically, ^1^H NMR chemical shifts are markedly sensitive to intermolecular NH⋅⋅⋅N and NH⋅⋅⋅O hydrogen bonding interactions which interconnect Hoogsteen faces, such that structurally different assemblies (herein quartet and ribbon‐like) exhibit distinct ^1^H DQ‐SQ spectral patterns, facilitating a direct identification of quartet and ribbon‐like assemblies.9c, 23 For example, ^1^H DQ‐SQ correlation NMR spectra have been presented for guanosine dihydrate (G⋅2H_2_O),23b isopropylidene‐guanosine (Gace)23c and 3′, 5′‐dipropanoyl deoxyguanosine dG(C3)_2_,23a in which there are crystal structures exhibiting different types of ribbon‐like self‐assembly. A ^1^H DQ‐SQ correlation spectrum has also been presented for a lyophilised guanosine borate hydrogel in the presence of K^+^ ions, whereby a ^1^H DQ “signature” (Figure 1 of ref. 9) of stacked G‐quartets exhibits a NH auto peak due to inter‐quartet stacking.

**Figure 4:**
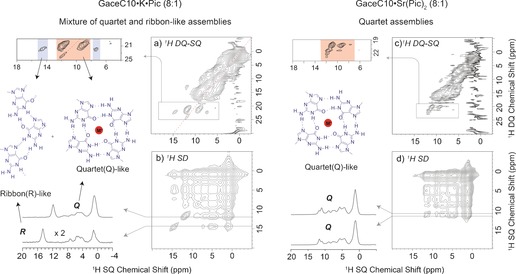
Two‐dimensional solid‐state ^1^H (850 MHz, 75 kHz MAS) NMR spectra of a GaceC10⋅K⋅Pic 8:1 complex (left) and a GaceC10⋅Sr⋅(Pic)_2_ 8:1 complex (right). (top) DQ‐SQ correlation spectra recorded using 1 *τ*_r_ of BABA recoupling^19^ and (bottom) NOESY‐type spin‐diffusion spectra (recorded with a mixing time of 106 ms) depicting the co‐existence of quartet and ribbon‐like assemblies for the GaceC10⋅K⋅Pic complex and only quartet assembly for the GaceC10⋅Sr⋅(Pic)_2_ complex. The base contour level is at: a) 0.6 %, b) 0.03 %, a) 0.8 % and d) 0.5 % of the maximum peak height.

Although complexing GaceC10 with 0.125 equivalents of K⋅Pic and Sr⋅(Pic)_2_ salts resulted in the formation of G4 assemblies in chloroform, only Sr⋅(Pic)_2_ fully retained G4 assemblies in the solid state. A two‐dimensional ^1^H DQ‐SQ correlation NMR spectrum of the GaceC10⋅K⋅Pic 8:1 complex is presented in Figure 4 a. The ^1^H DQ peaks at *δ*_DQ_ equal to 22.6 ppm (14.8+7.8) and 21.2 ppm (11.9+9.3) are assigned to intramolecular H−H proximities of NH1 and one of the NH2 protons in ribbon‐like and quartet assemblies, respectively.9c, 23 Moreover, as noted above,9c the NH1−NH1 autocorrelation peak at 23.8 (11.9+11.9) ppm is indicative of inter‐quartet stacking. A ^1^H NOESY‐like spin‐diffusion NMR spectrum of the GaceC10⋅K⋅ Pic 8:1 complex shown in Figure 4 b clearly reveals the co‐existence of both quartet and ribbon‐like arrangements. Specifically, no cross peaks are observed between the NH peaks assigned to quartet (*δ*_SQ_ between 10 to 12 ppm) and ribbon‐like (*δ*_*S*Q_ between 13 to 15 ppm) assemblies, even for spectra recorded for four different mixing times between 10 ms and 319 ms (Supporting Information, Figure S6). Thus, this spin‐diffusion MAS NMR data proves that the quartet and ribbon‐like peaks correspond to separate microcrystals within the powdered sample. Likewise, no cross peaks between quartet and ribbon‐like assemblies are observed in a ^1^H spin‐diffusion NMR spectrum for the GaceC10⋅KI 8:1 complex (Supporting Information, Figure S6). Although the ^1^H DQ and spin diffusion data of the K⋅Pic complex reveal the existence of separate quartet and ribbon‐like peaks, analogous spectra for a Sr⋅(Pic)_2_ complex showed only peaks corresponding to quartets within a single phase as indicated by a ^1^H spin‐diffusion NMR spectrum (Figure 4 d). We note that the formation of micro‐twinning structures such as those observed in metallohelicates/helicenes^24^ is not ruled out by the present study.

**Figure 5:**
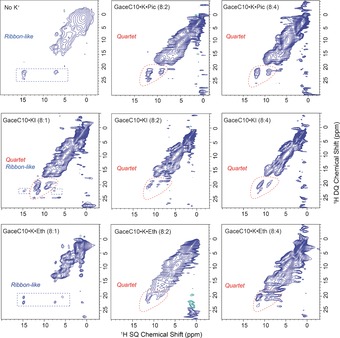
Two‐dimensional ^1^H (850 MHz, 75 kHz MAS) DQ‐SQ correlation solid‐state MAS NMR spectra of GaceC10 alone (top left) and GaceC10 complexes with K^+^ ions, recorded using 1 *τ*_r_ of BABA recoupling.^19^ Top: GaceC10, GaceC10⋅K⋅Pic 8:2 and 8:4 complexes; middle: GaceC10⋅KI 8:1, 8:2 and 8:4 complexes, and bottom: GaceC10⋅K⋅Eth 8:1, 8:2 and 8:4 complexes.

Figure 5 presents ^1^H DQ‐SQ correlation NMR spectra of GaceC10 in the absence and in the presence of K^+^ ions. G‐quartet based ^1^H DQ spectral patterns are observed in Figure 5 for the GaceC10⋅K⋅Pic 8:2 and 8:4, GaceC10⋅KI 8:1, 8:2 and 8:4 and GaceC10⋅K⋅Eth 8:2 and 8:4 complexes. For the GaceC10⋅K⋅ Eth 8:1 complex, the observation of two sets of ^1^H DQ peaks for the NH resonance at 14.8 ppm is likely due to proximity to both an NH2 and a CH8 proton (compare, for example, the ^1^H DQ spectrum of Gace presented in Figure 7 of ref. 23c).

Combining these observations from solution‐ and solid‐state studies, it can be inferred that: 1) solvent plays an important role in stabilizing the lipophilic G‐quartets when a smaller amount of potassium ions, typically less than 0.5 equivalents are used; 2) in the solid‐state, up to at least 0.5 equivalents of potassium ions (depending on binding anions) are essential to retain the structural integrity of G‐quartets. In solution, several factors are known to influence the kinetic and thermodynamic stability of G4 assemblies including the nature of solvent, concentration, pH and anion complexation: For example, Davis and co‐workers showed that organic anions bridge individual G‐quartets, much like clips holding the exteriors of G4 assemblies,12b, 14a whereas Meijer and co‐workers showed that solvent polarity regulates the formation of G4‐assemblies.14e In the solid state, consideration of X‐ray diffraction structures of analogous lipophilic G4 assemblies12b, 14a,14e suggests that 0.125 equivalents of cations ought to be sufficient for the formation of a lipophilic G‐quadruplex. Taken altogether, these observations encouraged us to examine the reversibility of quartet‐ribbon interconversions during the dissolution process.

### A dissolution NMR experiment demonstrates that quartet‐ribbon interconversion is reversible

**Figure 6:**
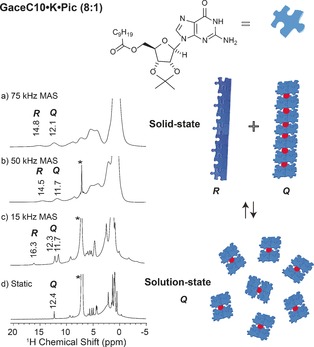
Single‐pulse ^1^H (850 MHz) MAS NMR spectra (left) depicting the solid‐state to solution‐state transformation of GaceC10⋅K⋅Pic 8:1 in CHCl_3_. a) 0.6 mg of powdered complex with no added solvent recorded using a JEOL 1 mm rotor at 75 kHz MAS, Q/R is 65:35, wt/wt. b) 1 mg complex in 2.5 μL CHCl_3_ (1 m solution) recorded using a Bruker 1.3 mm rotor at 50 kHz MAS, Q/R is 74:26, wt/wt. c) 0.5 mg complex in 5 μL CHCl_3_ (0.25 m) recorded using a 1.3 mm rotor at 15 kHz MAS, Q/R is 86:14 wt/wt. d) 0.25 mg complex in 5 μL CHCl_3_ (0.125 m) recorded under static conditions in a 1.3 mm rotor. A cartoon representation (right) depicting interconversion of a mixture of stacked quartet (Q) and ribbon (*R*)‐like structures into sandwiched quartet (Q) structures upon a solid‐state to solution‐state transformation.

Reversibility is a key feature in supramolecular chemistry. G‐derivatives that respond to external stimuli such as light, concentration, specific reagents, cation and anion binding are of considerable interest for designing switchable assemblies.9a, 14d, 25 To probe interconversion between quartet and ribbon‐like assemblies, powdered samples of GaceC10⋅K⋅Pic 8:1 were suspended in known quantities of CHCl_3_ and the dissolution process was monitored through a combined solution‐state/solid‐state NMR approach (Figure 6). We note that such a combined solution‐ and solid‐state NMR approach has previously enabled the investigation of labile chiral supramolecular ion pairs^26^ and monitored the crystallization process in small molecules.^27^

For the GaceC10⋅K⋅Pic 8:1 complex, Figure 6 presents ^1^H MAS spectra recorded during a dissolution experiment: the solid/solution ratio was 1 mg:2.5 μL, 0.5 mg:5 μL and 0.25 mg:5 μL for Figures 6 b–d, respectively. Note that the ^1^H MAS NMR spectra were recorded at different MAS frequencies as necessary to achieve good spectral resolution at the varying solid/solution ratios. Specifically, MAS frequencies of 75, 50 and 15 kHz were used for the dried powder, 1 mg sample in 2.5 μL chloroform (1 molar solution) and 0.5 mg of sample dissolved in 5 μL of chloroform (250 millimolar solution), respectively. A line shape fitting analysis showed that the Q/R ratio (error estimated as ±5 %) changes from 65:35 (Figure 6 a), to 74:26 (Figure 6 b), to 86:14 wt/wt (Figure 6 c), finally resulting in purely quartet‐like assemblies in chloroform, 0.125 m (Figure 6 d). This dissolution experiment clearly exemplifies the reversibility of quartet‐ribbon interconversions. The doubling of NH1 peaks between 11 to 12 ppm in Figure 6 c is likely due to the formation of a kinetically labile species, namely the *C*_4_‐symmetric octamer, as was similarly observed for the ^1^H solution‐state NMR spectra of the GaceC10⋅K⋅Pic 8:2 and 8:4 complexes shown in Figures 2 b and c. Note the peak with a high ^1^H chemical shift of 16.3 ppm in Figure 6 c: we hypothesise that this corresponds to a ribbon‐like intermediate mode of assembly, with distinct ribbon‐like structures having previously been observed.23b, 28 Considering all the solid‐state NMR results presented in this paper, we emphasise that only solid‐state NMR spectroscopy can provide this unique structural insight–by comparison, broad spectral features are observed in powder X‐ray diffraction patterns, as shown for example for the GaceC10⋅K⋅Pic 8:1 complex in Figure S4 (Supporting Information).

## Conclusions

To summarize, we have systematically investigated formation of distinct supramolecular assemblies for a long alkyl chain G derivative in chloroform and in the solid state. Solution‐state CD and NMR studies revealed the formation of G‐quartets in the presence K^+^ ions. Increasing the K^+^ ion concentration from 0.125 to 0.5 equivalents triggered the formation of a kinetically labile *C*_4_‐symmetric octamer that then slowly dissociated into a stable *D*_4_‐symmetric octamer, as monitored by time‐course NMR spectroscopy. GaceC10⋅K⋅Pic complexes exhibit relatively strong CD signals, compared to K⋅Eth and KI complexes, suggesting a more tightly bound complex for K⋅Pic. By comparison, 0.125 equivalents of Sr^2+^ ions induced the formation of a stable and long‐lived hexadecamer species in chloroform. Solution‐state PFG NMR data clearly showed that picrate anion strongly binds to the GaceC10⋅Sr^2+^ complex, but weakly binds to the GaceC10⋅K^+^ complex as revealed by measurable differences in the extracted diffusion coefficients (*D*) for both G‐quartets and picrate ions (see Table 1 and Figures 3 and S2). This can be explained by the stronger ion–dipole interactions between the oxygen atoms of Hoogsteen faces and the doubly charged Sr^2+^ ion.

Chemical intuition tells us that the adoption of a particular self‐assembled structure as driven by the formation of specific intermolecular hydrogen bonds in solution would be expected to lead to the persistence of the same mode of self‐assembly in the solid state. Intriguingly, this work reveals that this is not the case for the supramolecular assembly exhibited by a guanosine derivative in solution and in the solid state, with there being a subtle interplay between competing hydrogen‐bonding interactions and solvent and complexation effects that depend on both concentration and the actual cation and anion. Importantly, for dried powders prepared by evaporation of the specific solvents used, we have demonstrated the co‐existence of quartet and ribbon‐like supramolecular entities for GaceC10⋅K⋅Pic 8:1 and 8:2, and GaceC10⋅K⋅I 8:1 complexes. In future work, it could be interesting to see how changes to the protocols for preparing the dry solids (e.g., change of solvent or slurrying) affect the observed supramolecular self‐assembly. The observations in this work were enabled by the ability of ^1^H solid‐state NMR to “view” distinct intermolecular hydrogen‐bonding interaction, with the unusual mixed assemblies having been quantitatively characterized in the solid state by fast MAS ^1^H DQ and spin‐diffusion NMR experiments. Our work has further shown that ribbon‐quartet interconversion can be followed in a dissolution experiment, demonstrating reversibility upon a solid‐ to solution‐state transition and vice versa.

In conclusion, by employing a combined solution‐ and solid‐state NMR approach, it could be inferred that, alongside the crucial role played by solvent effects,14e higher salt concentration (typically ≥0.5 equiv K^+^ ions) is required to retain the structural integrity, and hence functionality of G‐quadruplexes in the solid state as compared to in solution. Otherwise, the solution‐ to solid‐state transition leads to mixed self‐assembly. This showcasing of the combination of solution‐ and solid‐state NMR reveals it to be a powerful approach for studying the formation of various other supramolecular assemblies in solution, gel and in the solid state.

## Further Experimental Data

See the Supporting Information for full experimental details including preparation of GaceC10 complexes, solution‐state time course NMR and DOSY spectra, CD spectra, powder X‐ray diffraction data, solid‐state NMR line shape analysis, ^1^H DQ‐SQ and spin‐diffusion MAS NMR spectra. Experimental data for this study is provided as a supporting dataset from WRAP, the Warwick Research Archive Portal at: http://wrap.warwick.ac.uk/84327.

## Supporting information

As a service to our authors and readers, this journal provides supporting information supplied by the authors. Such materials are peer reviewed and may be re‐organized for online delivery, but are not copy‐edited or typeset. Technical support issues arising from supporting information (other than missing files) should be addressed to the authors.

Supplementary
