# Supplementary material for: Co‐existence of Distinct Supramolecular Assemblies in Solution and in the Solid State
Source: Chemistry. 2016 Dec 22;23(10):2315–22. doi: 10.1002/chem.201604832 (PMC5396329; doi:10.1002/chem.201604832)
Supplement: Supplementary file 1 — Supplementary [file CHEM-23-2315-s001.pdf]

CHEMISTRY
A European Journal

Supporting Information

Co-existence of Distinct Supramolecular Assemblies in Solution and in the Solid State

G. N. Manjunatha Reddy,[a] Aida Huqi,[b] Dinu Iuga,[a] Satoshi Sakurai,[c] Andrew Marsh,[a] Jeffery T. Davis,[d] Stefano Masiero,[b] and Steven P. Brown*[a]

chem_201604832_sm_miscellaneous_information.pdf

Table of contents

1. Compound synthesis and sample preparation

2. Time course solution-state NMR

3. Circular Dichroism (CD) spectra of GaceC10 complexes

4. Powder X-ray diffraction spectra of GaceC10 and a GaceC10·K·Pic complex

5. Solid-state NMR line shape analysis

6. NOESY-like ^{1}H Spin-Diffusion (SD) MAS NMR spectra of GaceC10 complexes

1. Compound synthesis and sample preparation

(a) Preparation of 2',3'-O-isopropylidene-5'-decanoylguanosine, "GaceC10"[1]

To a stirred suspension of 2',3'-*O*-isopropylidene guanosine (3.55 g, 11 mmol, previously vacuum dried over P_2O_5 at 50 °C) and *N*,*N*-dimethylaminopyridine (0.10 g, 0.13 mmol, 0.1 eq) in acetonitrile (240 mL) in a dry three-necked round bottom flask was added decanoic anhydride (4.9 mL, 4.3 g, 13 mmol, 1.2 equiv.). The reaction was allowed to stir for 5 days at room temperature after which time thin layer chromatography (SiO_2, 5% MeOH:CH_2Cl_2, title product $R_f = 0.6$) indicated reaction completion. Methanol (25 mL) was added and the precipitate filtered under vacuum and the resultant solid washed three times with small amounts of acetonitrile, then water, then ethanol and finally acetonitrile to give a solid which was dried under vacuum overnight (3.56 g, 68%). This material was recrystallized from hot ethanol to give the *title product* as a white solid m.p. >250 °C; ^{1}H NMR (dmso-d_6) 10.7 (s, 1H), 7.84 (s, 1H), 6.60-6.40 (br., 2H), 6.00 (s, 1H), 5.25 (dd, 1H, *J* = 6.3, 1.9), 5.10 (dd, 1H, *J* = 6.2, 3.4), 4.24 (m, 2H), 4.12 (dd, 1H, *J* = 12.8, 8.0), 2.50 (m, ^{1}H-dmso-d_6), 2.25 (t, 2H) 1.47 (s, 3H), 1.45 (m, 2H), 1.32 (s, 3H), 1.25-1.21 (m, 12H), 0.85 (t, 3H) ppm; ^{13}C NMR (dmso-d_6) 172.7, 156.7, 153.7, 150.5, 136.2, 116.9, 113.3, 88.3, 84.2, 83.7, 81.1, 63.9, 40.1, 39.6, 39.1, 33.2, 31.2, 28.8, 28.7, 28.6, 28.4, 27.0, 25.3, 24.3, 22.1, 13.9 ppm (see Figure S1); ν_{max} (solid) 3443, 3304, 3169, 2921, 1746, 1706, 1636, 1597, 1541, 1486, 1383, 1211, 1156, 1064, 866, 780, 681 cm^{-1}; $C_{23}H_{36}N_5O_6$ (MH^+) requires *m*/*z* 478.2660. Found (ESI pos. mode) 478.2665. $C_{23}H_{35}N_5O_6$ requires C, 57.85; H, 7.39; N, 14.67%. Found C, 56.28; H, 6.99; N 15.94%. We note that obtaining "correct" elemental analysis of this compound has repeatedly proven problematic.

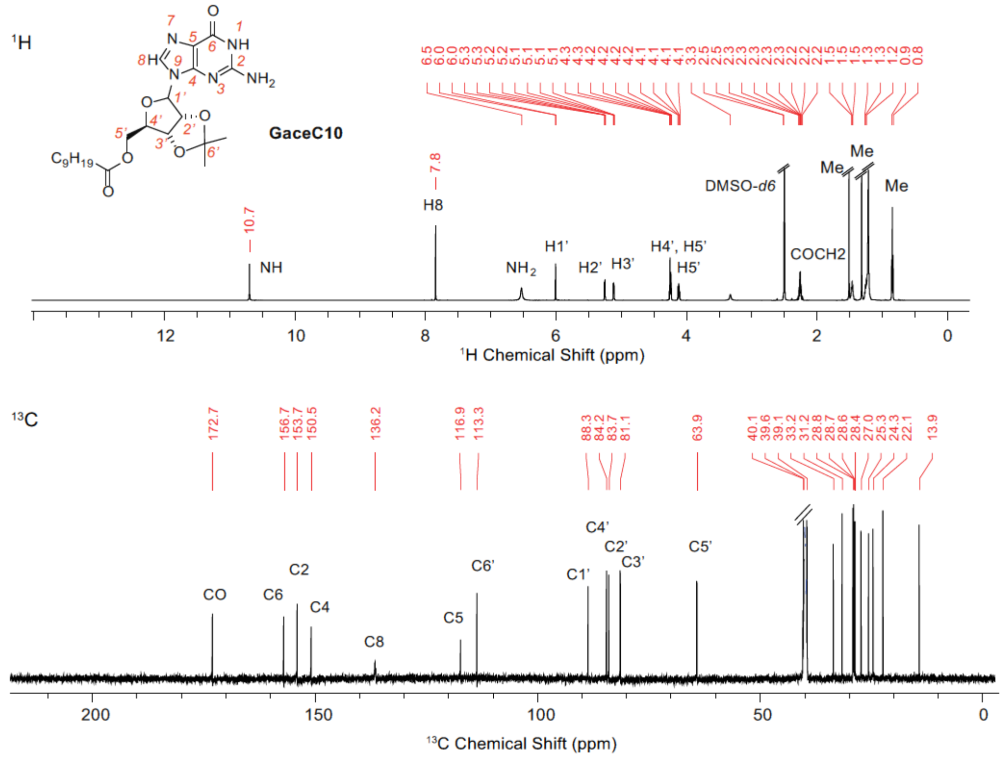

Figure S1: ^{1}H and ^{13}C one-pulse NMR spectra of GaceC10 in DMSO-d_6, recorded at a ^{1}H Larmor frequency of 600 MHz. For both ^{1}H and ^{13}C one pulse experiments, 4 and 11000 co-added transients were acquired, respectively.

(b) Preparation of the metal salt complexes of GaceC10 (see Scheme S1)

For picrate complexes, the appropriate molar ratio of the potassium or strontium salt, dissolved in acetonitrile (0.1-0.2M), was added to a stirred solution of GaceC10 (150 mg) in chloroform (10 mL) and the sealed vial containing the mixture was stirred for 1 hr at r.t. For ethanoate and iodide complexes, the appropriate molar ratio of the solid potassium salt was added to a stirred solution of GaceC10 (150 mg, 0.31 mmol) in chloroform (10 mL) and the sealed vial containing the mixture was stirred at r.t. for up to 14 days to allow complete dissolution. In all cases, the vial was then opened, protected from dust, and the solvent was allowed to evaporate at r.t. and atmospheric pressure. GaceC10-KEth and GaceC10-KI complexes were recrystallized by using hot ethanol or ethylacetate. The resultant solution was left to cool down to room temperature and solvent was allowed to slowly evaporate at r.t. over 2 days in a round bottomed flask protected from dust. Finally, rotary evaporation was performed at approximately 5 mm Hg pressure for a gentle removal of any residual solvent molecules. The dried powders were subjected to solid-state NMR spectroscopy measurements.

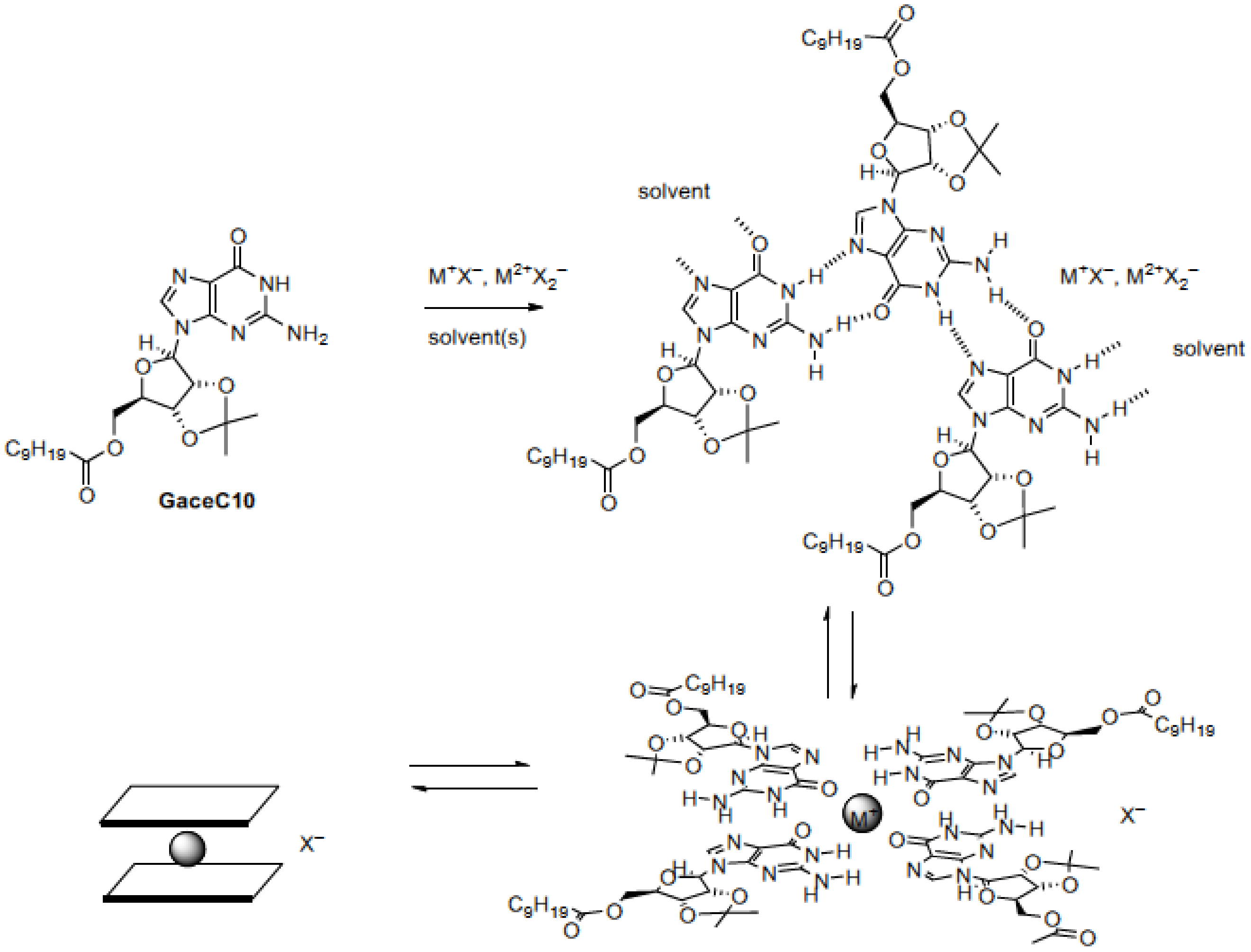

Scheme S1: GaceC10 complexation with metal ion salts.

2. Time course solution-state NMR

For solution-state NMR spectra of GaceC10·K·Pic 8:2 and 8:4 complexes recorded immediately after dissolution, integration for the NH peaks revealed that the relative proportions of major species (δ_{NH}, 12.38 ppm) and minor species (δ_{NH}, 11.41 and 11.82 ppm, 1:1) were 83/17 and 87/13, respectively. Minor octamer species were then observed to slowly rearrange into major species that exhibit a single set of peaks in their NMR spectra recorded 8 h after dissolution (see Figure S2). Solution-state pulsed field gradient (PFG) NMR experiments were performed immediately after sample dissolution and 8 h after dissolution using a Bruker Avance 500 NMR spectrometer (see Figure S2). Temperature control was set to 298 K during the time course NMR data acquisition. DOSY data were acquired using a bipolar-paired gradients incorporated with a longitudinal eddy current delay (BPP-LED) sequence.[2] PFG NMR data were acquired with 16 co-added transients, with a total diffusion encoding pulse duration, δ, of 2 ms and a diffusion delay, Δ, of 120 ms by using 16 gradient amplitudes ranging from 0.7 G cm^{-1} to 25.5 G cm^{-1}. 2D data were processed using the DOSY toolbox[3] by fitting the data to the mono-exponential Stejskal-Tanner equation:[4]

$$S_{(g)} = S_0 \exp(-D\gamma^2\delta^2 g^2 \Delta') \,, \qquad \text{(S1)}$$

where $S_{(g)}$ and S_0 is the echo amplitude with and without applied gradients, respectively, γ is the gyromagnetic ratio, δ is the duration of the gradient pulses, and $\Delta' = (\Delta - \delta/3)$ is the effective diffusion encoding time, where Δ is the time interval between gradient pulses. Diffusion coefficients measured immediately after dissolution and 8 h after the dissolution are listed in Table 1 (main text) for G4 assemblies and picrate ions.

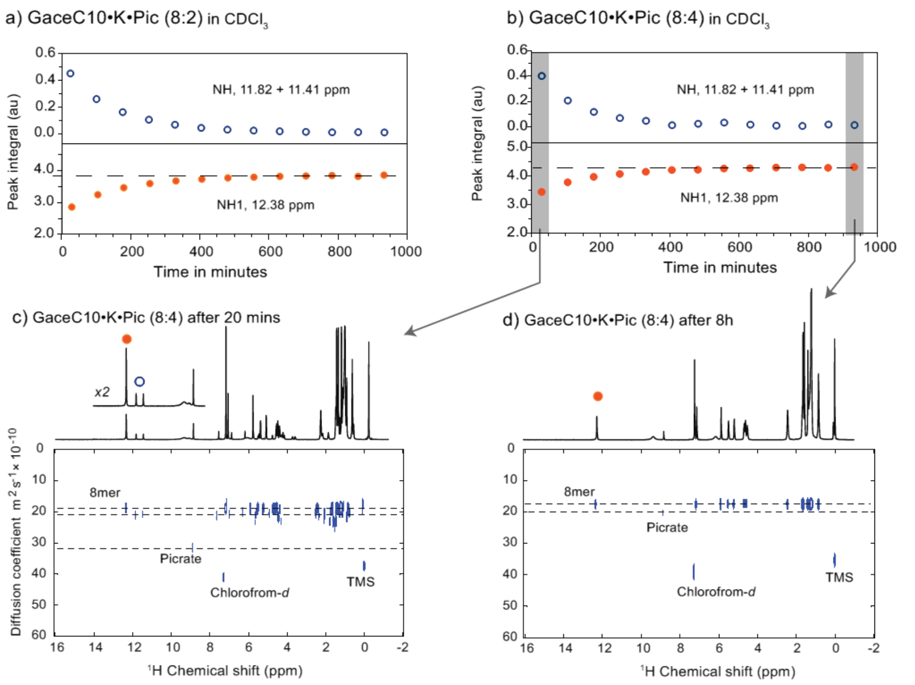

Figure S2. Top: integrals of NH peaks (closed orange circles correspond to 12.38 ppm, while open blue circles correspond to 11.82 and 11.41 ppm) are plotted as a function of time for 32 mM GaceC10·K·Pic (a) 8:2 and (b) 8:4 complexes in chloroform-d. Time course NMR (500 MHz) experiments were initiated immediately after the dissolution and acquired overnight. Bottom: ^{1}H (500 MHz) DOSY NMR spectra of a GaceC10·K·Pic 8:4 complex, 16 mM in $CDCl_3$, acquired (c) immediately after dissolving in $CDCl_3$ and (d) 8 h later.

3. Circular Dichroism (CD) spectra of GaceC10 complexes

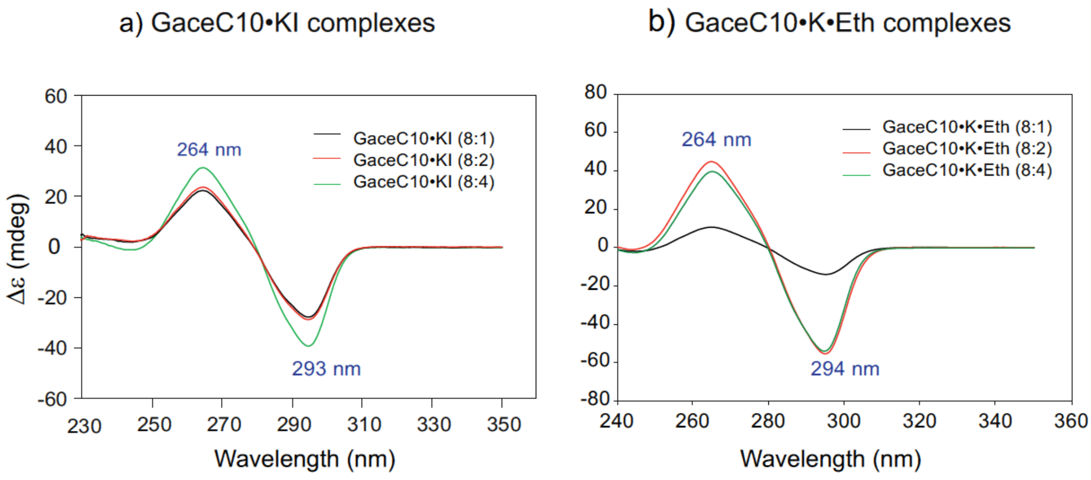

Figure S3. CD spectra in chloroform, 0.65 mM, for (a) GaceC10·KI and (b) GaceC10·K·Eth complexes.

4. Powder X-ray diffraction (PXRD) spectra of GaceC10 and a GaceC10·K·Pic complex

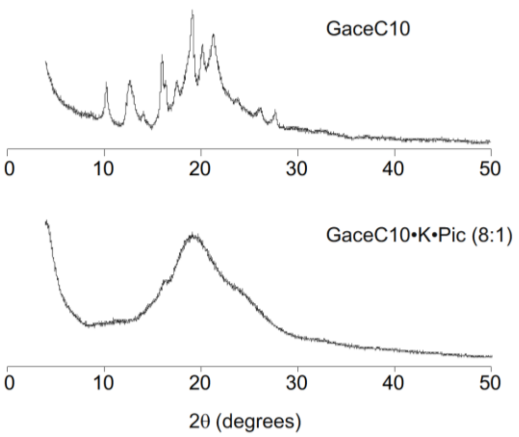

Figure S4. Powder X-ray diffraction patterns for GaceC10 (top) and a GaceC10·K·Pic (8:1) complex (bottom).

5. Solid-state NMR line shape analysis

NMR line shape analysis (see Figure S5) was performed using the line shape tool implemented in Bruker Topspin 2.1. A fitted envelope with mixed Lorentzian and Gaussian shapes was used for NH1 peaks between 10 ppm to 16 ppm to extract the peak integrals. Errors were estimated to be of the order of 5% (i.e., varying the peak integral values by 5% resulted in no significant change in the fitted line shapes). The relative proportion of quartet and ribbon-like assembly is calculated on the basis of the deconvoluted peak integrals.

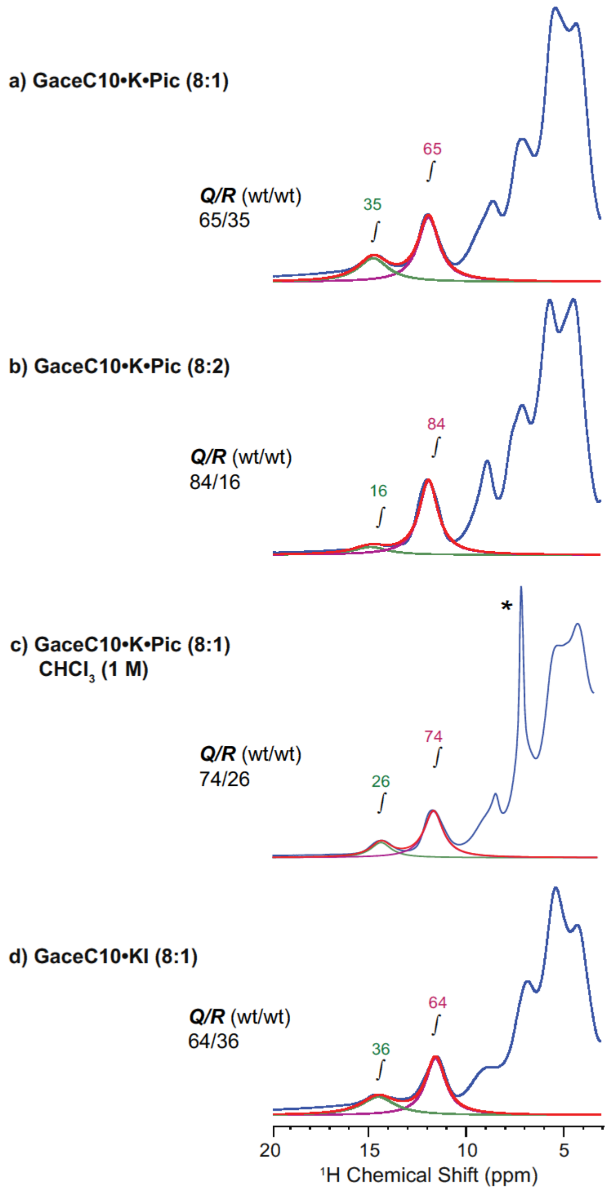

Table of fitting parameters

	*NH1(**R**)*	*NH1(**Q**)*
δ_{iso} (ppm)	14.8	12.1
Lorentzian broadening (Hz)	1520	1100
Gaussian broadening (Hz)	200	100

δ_{iso} (ppm)	14.8	12.1
Lorentzian broadening (Hz)	2000	950
Gaussian broadening (Hz)	100	200

δ_{iso} (ppm)	14.8	12.1
Lorentzian broadening (Hz)	1000	1000
Gaussian broadening (Hz)	500	200

δ_{iso} (ppm)	14.6	11.4
Lorentzian broadening (Hz)	1750	1000
Gaussian broadening (Hz)	300	400

Figure S5. Line shape fitting analysis of single-pulse solid-state ^{1}H (850 MHz) MAS NMR spectra of (a) GaceC10·K·Pic 8:1, (b) GaceC10·K·Pic 8:2, (c) a 1M solution of the GaceC10·K·Pic 8:1 complex in $CHCl_3$, and (d) GaceC10·KI 8:1. These correspond to the spectra presented in Figures 2a, 6b, 2b and 2e in the main text. In all cases, experimental spectra are depicted in blue and the red spectra are the fitted envelopes of deconvoluted ribbon-like (green) and quartet-like (magenta) NH1 peaks. Relative amounts of Quartet (Q) and Ribbon(R)-like assemblies by wt% are Q/R, 65/35, 84/16, 74/26 and 64/36 respectively in (a), (b), (c) and (d). Parameters used for the line shape analysis are reported in Tables adjacent to the NMR spectra.

6. NOESY-like ^1H Spin-Diffusion (SD) MAS NMR spectra of GaceC10 complexes

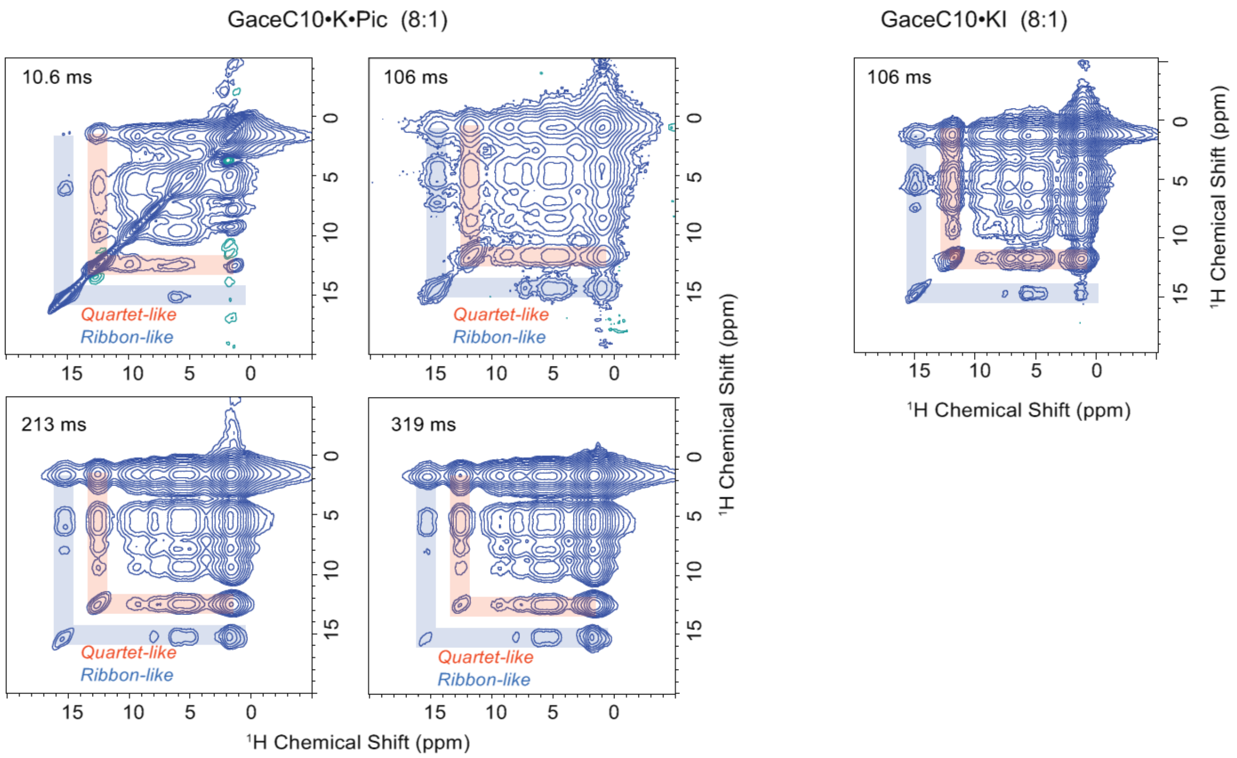

Figure S6. Two-dimensional ^1H (850 MHz, 75 kHz MAS) NOESY-type spin-diffusion solid-state MAS NMR spectra of a GaceC10·K·Pic 8:1 complex (left, recorded at variable mixing times) and a GaceC10·KI 8:1 complex (right, recorded using a mixing time of 106 ms). No cross peaks are observed between NH resonances corresponding to ribbon-like (shaded in blue) and quartet (shaded in red) assemblies, indicating that ribbon-like and quartet GaceC10 assemblies are separate microcrystals in the powdered sample.

Acknowledgements

We thank Dr Lijiang Song (Department of Chemistry, University of Warwick) for help and advice with mass spectrometry, Dr Ivan Prokes (Department of Chemistry, University of Warwick) for assistance and acquiring PFG and DOSY NMR spectra, and Dr David Walker (X-ray diffraction Research Technology Platform, University of Warwick) for assistance with PXRD measurements.

References

[1] a) T. Giorgi, F. Grepioni, I. Manet, P. Mariani, S. Masiero, E. Mezzina, S. Pieraccini, L. Saturni, G. P. Spada and G. Gottarelli, *Chem. –Eur. J.* **2002**, *8*, 2143-2152; b) I. Manet, L. Francini, S. Masiero, S. Pieraccini, G. P. Spada and G. Gottarelli, *Helv. Chim. Acta* **2001**, *84*, 2096-2107; c) S. Lena, M. A. Cremonini, F. Federiconi, G. Gottarelli, C. Graziano, L. Laghi, P. Mariani, S. Masiero, S. Pieraccini and G. P. Spada, *Chem.—Eur. J.* **2007**, *13*, 3441-3449.
[2] D. H. Wu, A. D. Chen and C. S. Johnson, *J. Magn. Reson, Series A* **1995**, *115*, 260-264.
[3] M. Nilsson, *J. Magn. Reson.* **2009**, *200*, 296-302.
[4] E. O. Stejskal and J. E. Tanner, *J. Chem. Phys.* **1965**, *42*, 288-299.
